# Is there a better evolutionary outcome in a 4-cell tetrahedron embryo?

**DOI:** 10.5935/1518-0557.20210034

**Published:** 2021

**Authors:** Marta Ribeiro Hentschke, Ricardo Azambuja, Victória Campos Dornelles, Bibiana Cunegatto, Cristina Hickman, Rishabh Hariharan, Isadora Badalotti Telöken, Catarina Heckmann Petracco, Fabiana Mariani Wingert, Alvaro Petracco, Mariangela Badalotti

**Affiliations:** 1 Fertilitat - Reproductive Medicine Center, Porto Alegre, Brazil; 2 Apricity - Virtual Fertility Clinic, London, England

**Keywords:** blastocyst development, irregular cleavage, morphokinetics, planar embryos, tetrahedron embryos, time-lapse imaging

## Abstract

With the growing understanding of in vitro fertilization and reproductive technology, the magnitude of studies related to embryonic evolution has also increased. The optimization of embryo selection is crucial to minimize the risk of multiple pregnancies and to guarantee successful implantation and pregnancy. On the second day of culture, the four-cell embryo can be shaped into different arrangements, such as tetrahedral and planar. Previous studies have shown that mammalian embryos have a tetrahedral shape and that any deviation from this ideal configuration can negatively affect blastocyst development. A few studies have also found that planar embryos would be linked to negative predictors of success for reaching the blastocyst stage and its good quality. Therefore, it seems that tetrahedral should be preferred over planar-shaped embryos for embryonic transfers, but there is still little understanding and evidence about this subject. Thus, the objective of the present paper was to review the available literature on study tendencies to compare tetrahedral and planar-shaped embryos considering their effect on implantation and pregnancy results.

## SEARCHING FOR THE PERFECT EMBRYO

Shortly after Louise Brown's birth, ^Steptoe *et al*. (1980)^ showed that in vitro fertilization (IVF) success rates were 6% per cycle. Today, this represents less than a fifth of the success rates offered with current reproductive technology. Over the years, greater knowledge about gametes' physiology and their behavior, as well as improved laboratory conditions, have yielded better embryonic potential and fertilization rates.

Despite these significant advances, there remains a growing interest in analyzing the predictive ability of specific events occurring during embryonic evolution to improve both embryonic implantation and clinical pregnancy rates. It is this constant desire for improved outcomes and an increased emphasis on minimizing the risks of multiple pregnancies that stresses the importance of optimizing the embryo selection stage - a stage which is highly compatible with the implementation of artificial intelligence (AI) ^(Figueira, 2017)^.

## THE "DIGITAL ERA"

The dawn of the "Digital Era" has illuminated the potential for mathematical algorithms associated with complex operating systems to accurately identify the best embryo models and, therefore, the best implantation rates. The implementation of AI into a time-lapse operating system may enable a constant evaluation of morphological characteristics throughout the embryonic development, using parameters (e.g. pixel values) that are otherwise inconspicuous to the human eye. This allows for the possibility of an AI and time-lapse system capable of embryo classification. However, it is still unknown whether there is a positive impact on using AI, since the difference in the correlation between the choice of embryo made by embryologists and pregnancy rates is still not well established in the literature ^(Gardner *et al*., 2015)^.

## THE FOUR-CELL EMBRYO - PLANAR OR TETRAHEDRAL DEVELOPMENT

The Istanbul consensus of ^Alpha and ESHRE (2011)^ established a series of standards for the evaluation of embryonic quality, intending to provide better results for assisted reproduction laboratories worldwide (Istanbul Consensus, 2011). The four-cell embryo is expected on the second day of development (D2), 44 (±1) hours after the intracytoplasmic sperm injection (ICSI) procedure. A good quality 4-cell embryo must present four identically sized blastomeres, absence of anucleate fragments or cytoplasmic changes, and the presence of visible nuclei in each of the blastomeres ^(Figueira, 2017)^.

Among the embryonic factors under study, the embryonic cells' arrangement, when the embryo presents itself with a count of four blastomeres, has been gaining increased attention. ^Gulyas (1975)^ demonstrated that rabbit 4-cell embryos might be arranged in a tetrahedral or non-tetrahedral arrangement, one of these later described as planar ^(Gulyas, 1975; Ebner *et al*., 2012)^.

To reach the 4-cell stage, 2-cell embryos can follow two distinct cleavage patterns. The direction of these divisions interferes with the cells' position and the spatial arrangement of blastomeres ^(Bischoff *et al*., 2008)^. Cleavage in both meridional and equatorial axes leads to a tetrahedral 4-cell configuration (3 blastomeres at the "bottom" plane and one at the top), whereas cleavage in only one axis leads to a planar constellation (4 adjacent blastomeres) ^(Ebner *et al*., 2012; Ajduk *et al*., 2014; Bischoff *et al*., 2008)^. However, what could influence planar or tetrahedral development, and its outcomes related to ART, is still under study.

^Graham & Lehtonen (1979)^ observed that offspring cells in the second cleavage of zone-free embryos in the 4-cell stage were generally moved, resulting in a variety of arrangement patterns in the conclusion of cell division. These patterns were classified according to the total number of cell-to-cell contacts, made within each embryo. Embryos organized in a tetrahedral manner enable greater intracellular contact between all blastomeres when compared to those in planar formations. Furthermore, ^Suzuki *et al*. (1995)^ reported in a mice study that the birth rates of embryos without a zona pellucida appeared to be influenced not only by their inherited phenotypic characteristics, but also by their relative cell-to-cell position in the 4-cell stage.

The tetrahedral configuration results in six intercellular contacts, whilst the planar embryo, formed with four adjacent blastomeres, can only have four ^(Ebner *et al*., 2012)^. The process of embryo compaction occurs more easily when there is an approach blastomere and increased cell adhesion ^(Sozen *et al*., 2014)^. In planar embryos, the mitotic spindle might have been affected, e.g. sperm centrosome composition or function, which in turn might have led to an abnormal cleavage ^(Ebner *et al*., 2012)^.

**Figure 1:**
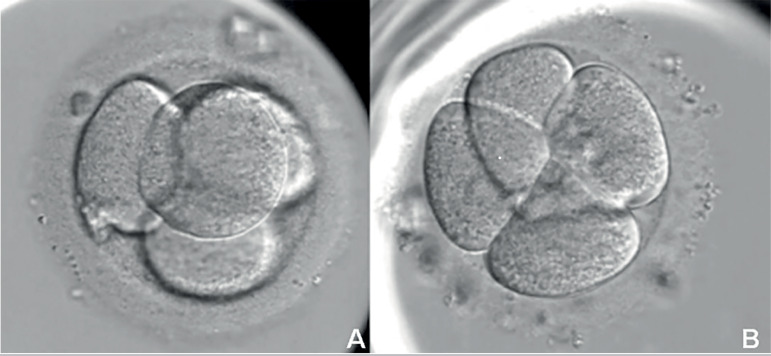
4-cell embryo on the second day of development A. tetrahedral arrangement embryo. B. planar arrangement embryo.

Figure 1 shows the microscopic comparison of the 4-cell embryo on the second day of development.

## TETRAHEDRAL *VERSUS* PLANAR EMBRYO OUTCOMES

Literature regarding the use of the 4-cell embryo conformation as a factor for embryo development is very much in its infancy, but there has been significant findings within this short span.

A study analyzing planar embryos, performed by ^Ebner *et al*. (2012)^, found that its prevalence was lower during conventional IVF, when compared to ICSI and cases of testicular sperm extraction. The planar arrangement was observed in 3.1% (64/2070) of embryos. Interestingly, these planar embryos showed better morphology on day 2 and day 3 than their sibling tetrahedral counterparts, but embryo blastocyst formation and implantation rates were higher in tetrahedral embryos than in planar embryos. None of the 11 planar embryos that were transferred was implanted. However, this result did not reflect differences in overall implantation and gestational rates. The risk of facing the planar cleavage pattern was associated with higher estradiol levels and the number of cumulus-oocyte-complexes.

^Cauffman *et al*. (2014)^ found that tetrahedral-shaped 4-cell stage embryos had a higher chance to blastulate and formed significantly higher quality blastocysts when compared to non-tetrahedral embryos. Moreover, tetrahedral embryos also had a higher chance to develop into high-quality embryos on days 3 and 5, while non-tetrahedral embryos showed a higher risk of arrest at these cleavage stages.

Concurring to these findings, ^Paternot *et al*. (2014)^ compared tetrahedral embryo transfer to the non-tetrahedral arrangement embryo transfers and found higher implantation rates (38% vs. 21%; *p*=0.038), pregnancy rates (33% vs. 16%; *p*=0.032), and live birth rates (33% vs. 16%; *p*=0.032) in the tetrahedral group, stating the importance of including this characteristic in embryo evaluation before transfer. Furthermore, in line with ^Cauffman *et al*. (2014)^, this study showed significant better development outcomes regarding the tetrahedral embryos, with a more frequently development into good-quality embryos (67% *vs*. 38%; *p*<0.0001), and excellent-quality embryos (42% *vs*. 19%; *p*<0.0001), as well as a higher development rate into 8-cell stage embryo on day 3 (45% *vs*. 24%; *p*<0.0001), which was different from the findings of ^Ebner *et al*. (2012)^ regarding embryo quality. As a limitation, this was a retrospective study that did not evaluate the molecular mechanisms behind its results; however, they analyzed a larger number of 4-cell-stage embryos than some of the previous similar studies.

To highlight the importance of intercellular contact for embryo outcomes, ^Liu *et al*. (2015)^ investigated this matter regarding good-quality 4-cell human embryos in a time-lapse study, including implantation outcomes. From 765 4-cell embryos analyzed, 33.8% presented fewer than six intercellular contacts at the end of this development stage, which resulted in worse embryo outcomes, such as worse conventional embryo scores and lower implantation rates after embryo transfer. Moreover, ^Paternot *et al*. (2014)^ showed the higher number of intercellular contacts in the 4-cell embryo conformation, associated with a greater development into an 8-cell stage embryo. In addition, in their analysis, the fewer intercellular contacts occurrence was not associated with female age and protocols performed. However, it was associated with a higher number of aspirated oocytes and estrogen levels, in agreement with ^Ebner *et al*. (2012)^. This higher ovarian stimulation association may be a reason why supraphysiological estrogen levels are associated with low pregnancy rates in the literature, reinforcing how a better understanding of embryo morphokinetics could improve assisted reproductive techniques in many aspects. However, this study was retrospective and excluded the poor-quality embryos from the intercellular contact analysis, which could interfere in its interpretation.

In another study by ^Ebner *et al*. (2017)^, using a time-lapse system, the authors reported again that tetrahedral embryos were more likely to survive to the blastocyst stage, and even showed better quality than planar embryos. They also reported that embryos with 4-cells and a planar arrangement would have negative predictors of success, already showing a delay in pre-implantation development. Additionally, they found that the viable blastocysts derived from D2 planar embryos would be more likely to consist of aneuploid cells. As previously discussed, this study highlighted that irregularities in the mitotic spindle and/or function of the spermatic centrosome might cause conformational issues. This indicates that planar embryos are rather abnormal, and the authors suggested that these embryos should only be considered for transfer if no other embryos are available, in line with the conclusions by ^Paternot *et al*. (2014)^.

More recently, in 2019, in agreement with previous studies, a better prognosis for tetrahedral embryos was found and the non-tetrahedral embryos resulted in less top-quality blastocysts, with a lower ability to blastulate, a higher frequency of dysmorphism, multinucleation, and irregular division. The results from ^Desai & Gill (2019)^ showed that a higher percentage of tetrahedral embryos met critical developmental benchmarks, such as compaction, morula formation, and developing to an extended blastocyst, leading to higher chances of cryopreservation/transfer when compared to non-tetrahedral embryos (OR, 3.58; CI, 2.42-5.28; *p*<0.001) and to planar embryos (OR, 4.20; CI, 2.13-8.29; *p*<0.001). However, the implantation, pregnancy, and live birth rates were similar in both groups, but this study has the limitation of being retrospective and nonrandomized.

## CONCLUSION

Identifying embryos with the highest implantation potential and the likelihood of live birth has long been the goal of embryologists worldwide. The spatial arrangement of blastomeres may play a critical role in embryo development. Animal, and subsequently, human embryo research has shown that mammalian embryos typically have a tetrahedral shape. Evidence increasingly shows that any deviation from this configuration may decrease the number of intracellular contacts, which is considered of extreme importance for cellular compacting and blastocyst development ^(Ebner *et al*., 2012; Graham & Deussen, 1978; Suzuki *et al*., 1995)^.

In view of the "Digital Era", with better accuracy to assess cell distribution at the exact moments of embryonic development through time-lapse technology, it seems to be of great importance to value the choice of the embryo based on its cellular configuration on the second day of development. When in the face of the choice between a blastocyst from a tetrahedral D2 embryo and a planar D2 embryo, based on the studies presented so far, the former would be the most suitable option for embryo transfer.

A better understanding of what may potentially influence the formation of a 4-cell embryo may prove critical for the future of IVF, as it is highly likely that this will become a valuable predictive factor for choosing the best embryo for embryonic transfer, and therefore improving pregnancy outcomes.
